# Person–Job Misfit: Perceived Overqualification and Counterproductive Work Behavior

**DOI:** 10.3389/fpsyg.2022.936900

**Published:** 2022-07-22

**Authors:** Jawad Khan, Amna Ali, Imran Saeed, Alejandro Vega-Muñoz, Nicolás Contreras-Barraza

**Affiliations:** ^1^Department of Business Administration, Iqra National University, Peshawar, Pakistan; ^2^Institute of Business and Management Sciences, The University of Agriculture, Peshawar, Pakistan; ^3^Public Policy Observatory, Universidad Autónoma de Chile, Santiago, Chile; ^4^Facultad de Economía y Negocios, Universidad Andres Bello, Santiago, Chile

**Keywords:** perceived overqualification, person–job (mis)fit, counterproductive work behavior, job boredom, job crafting

## Abstract

Grounding on person–job fit theory, we examined perceived overqualification relation with counterproductive work behavior (CWB) by identifying job boredom as a mediator and job crafting as a moderator. Hierarchical linear regression and Hayes’ PROCESS macro-method were used to assess hypotheses in a three-wave survey of 317 textile sector employees. The findings show that perceived overqualification is positively related with CWBs. This study further examined the mediating function of job boredom and the moderating impact of job crafting in the association between perceived overqualification and CWB. The findings suggest that job crafting moderates the positive relation between perceived overqualification and job boredom and the indirect connection between perceived overqualification and CWB *via* job boredom. The model was tested using 3-wave data; however, since the data were attained from a single source, questions of common method bias cannot be ruled out. Managers should look for changes in employee attitudes and promptly modify employees’ positions when they indicate that they have more experience, abilities, and talents required for their roles in their organizations.

## Introduction

Qualified employees are the key sources that make organizations sustainable and competitive. The organization should provide the employees with an environment where they can flourish. The jobs should have the skill variety, job identity, and job significance as Hackman and Oldham claimed in their job characteristics model so that employees find them meaningful and motivating (De Sousa Sabbagha et al., 2018; Erdogan and Bauer, 2021). It has been recognized in human resource management (HRM) literature that employees who think they are overqualified are a kind of human capital (Sesen and Ertan 2020). Researchers have identified employee development programs are valuable tools to efficiently use overqualified employees to achieve organizational objectives. When this “demand and supply” interacts, holding exogenous factors constant, the internal part of the success (regarding employees) will be achieved. But this is not what always happens. The discrepancy may arise either from the employee or organization or sometimes much extensively from cultural ideas, economic situations or employment policies.

A satisfactory amount of evidence reveals that employees are more qualified than their jobs’ demands (Brynin, 2002; Arvan et al., 2019). For example, in different countries such as United Kingdom, Canada, United States of America and Germany the one-third of employees are overqualified. Similarly, this ratio was 39% in the general EU zone according to CEDEFOP’s (The European Centre for the Development of Vocational Training) research in 2018 (Gkorezis et al., 2019), 84% in China, and 47% worldwide (Huang and Hu, 2022). For (Howard et al., 2022) the reason is the increasing competition in the labor market and the downturn of the global economy. With the Covid-19 pandemic, this problem plays a more substantial role in societies (Gong et al., 2021). Qualified individuals contribute to the performance and creativity of an organization as a result of holding extensive qualifications (Erdogan et al., 2011). In contrast, overqualified workers are more likely to have negative work attitudes and may not be willing to exert additional effort. This is why the overqualification problem has drawn the attention of academics, practitioners, and business leaders (Maynard and Feldman, 2011). According to previous studies, individuals and their employers may suffer from overqualified employees.

Previous studies suggest that POQ has negative outcomes for both employees and organizations. POQ is linked with low job satisfaction (Wassermann et al., 2017), demotivation (Chambel et al., 2021a), frustration, and impaired health (Liu et al., 2015). Studying previous literature, still, there is an important gap in the POQ literature regarding withdrawal behaviors, and the psychological nature of the POQ. Furthermore, unpacking when, why, and how POQ exhibits such behavior? The researcher starts examining the links between POQ and employee behavior, such as withdrawal behavior. One such important behavior is counterproductive work behavior (CWB)—which refers to “voluntary acts that are detrimental to an organization’s goals that contradict important organizational rules and endanger its well-being or its members” (Bakker et al., 2012). Due to poor person–job fit, such relation is important to be examined that triggers employee CWB. Moreover, a model with mediating and moderating paths between POQ and CWB is still underdeveloped. So it is important to explain when, how and why POQ employees show CWB’s. However, it has been shown that the relationship between perceived overqualification and CWBs is caused by negative emotions and behaviors that emerges due to a poor needs-supplies fit, stressing the significance of satisfying needs in the regulation of perceived overqualification (Gong et al., 2021). This results in a negative emotions and behaviors, which leads to CWBs. In line with the person–job fit theory and expanding it further, we argue that for employees who see themselves as overqualified, enhancing the needs–supplies fit may encourage productive work behavior (Erdogan and Bauer, 2021).

Person–job misfit is one of the reasons behind job boredom at the workplace. Research on this topic is scarce compared to employees with surplus knowledge, skills, and abilities (KSA’s) and a lack of challenging tasks and responsibilities. These beliefs substantially influence employees’ attitudes and emotions about their job qualification (Seong et al., 2015). When people’s working settings do not correspond to their interests or expectations, they are more prone to experience feelings of boredom (Fisher, 1993). Individuals’ beliefs that they are overqualified for a position or a mismatch between their education, experience, abilities, and responsibilities required in a position (for example, individuals may believe that they are overqualified for a position) may play a role in the boredom that they experience in their jobs (Liu and Wang, 2012). Employees may actively alter their work better to meet their needs, talents, and motivations when this form of misfit emerges. They try to engage in such behaviors that is make their job interesting. One such approach is job crafting, “Job crafting is an individually driven work design process which refers to self-initiated, proactive strategies to change the characteristics of one’s job to better align the job with personal needs, goals, and skills.” However, such technique is important to examine whether job crafting lower the effect between perceived overqualification and job boredom in the workplace. Previous studies find a positive relation between employee’s surplus knowledge, skills and abilities (KSAs) required for a job and their passion toward making job interesting (Tims and Bakker, 2010).

This study shows that the person–job fit theory is an important theoretical approach to theorizing overqualification. Person–job fit theory highlights various behavioral consequences due to such mismatch. Grounding on person–job fit theory, we empirically examined overqualification with key behavioral outcomes “counterproductive work behavior.” Our study shows that POQ is positively linked with harmful behavior and showing that CWB is a result of a person–job mismatch (Sánchez-Cardona et al., 2020). Further, this study also contributes to the job crafting literature by examining it mitigating role in reducing job boredom due to POQ. Job boredom starts when an employee qualification does not align with job requirements and responsibilities or there is a poor mismatch between needs-supplies fit. This study is evidence to examine job crafting as a buffering mechanism in predicting relation between POQ and job boredom. This study shows that self-initiated and proactive strategies helps employees to reduce job boredom among overqualified employees.

## Hypotheses Development

### Perceived Overqualification as Person–Job Misfit

Overqualification is defined as “individuals possessing extra knowledge, skills, and abilities (KSAs) in comparison with the requirements of their jobs” (Liu et al., 2015). The person–job fit framework is the one that most accurately expresses the essence of excessive qualification. Perceptions of surplus qualifications are aligned with the concept of person–job mismatch, which refers to when people’s talents exceed work requirements (i.e., demands–abilities mismatch). Furthermore, this approach highlights the demands of these employees are being met (i.e., needs–supplies mismatch). Employees with surplus qualifications may be able to carry out their essential responsibilities. Still, they are unable to use the talents they have acquired, and as a result, they are likely to be underutilized, resulting in increased stress and boredom (Edwards, 1991). Overqualification may be quantified empirically, such as when comparing education and experience levels required for a specific job position (Kristof-Brown et al., 2005).

Similarly, individual perceptions of overqualification are the most reliable means of determining it. (i) It is often a position taken on the basis of social comparison, and (ii) the norm against which qualifications are assessed is typically subjective (Liu and Wang, 2012). The subjective comparisons that people make between their current employment and standard benchmarks such as their self-esteem, former job, and coworkers in comparable positions serve as the basis for overqualification and influence employee behavior. In addition, studies show that applicants’ perceived and empirically judged overqualifications are strongly linked (McKee-Ryan et al., 2009). Our focus in this study is on the perception of overqualification due to these difficulties in line with past research. Person–job fit literature illustrates, a mismatch always results in negative consequences, when it comes to high performance, well-being, and favorable attitudes toward their work, fit is valuable for both individuals and organizations (Edwards, 2008). According to this theory, employees who are overqualified is more likely to be unhappy with their job and less committed to their work (Edwards, 2008; Wu and Chen, 2021).

Furthermore, previous research specify that employee who are overqualified are more inclined to engage in more job-seeking activities and deviant behavior. Overqualified individuals are engaged in various activities, and there has been little research into why they are doing so and what factors help mitigate these actions’ negative consequences. The role of moderators and mediators in influencing behavioral outcomes of the person–job mismatch is unclear and less studied (Edwards, 1991; Erdogan and Bauer, 2009).

### Perceived Overqualification and Job Boredom

The feeling of job boredom is widespread, yet it has been a neglected research topic. Job boredom is a depressing situation characterized by low arousal levels and displeasure, a distorted sense of time, and concentration issues, and is usually connected with insufficient stimulus in the workplace (Kim et al., 2021). Many studies have focused on work-related characteristics as predictors of boredom. Repetitive tasks and monotony factors may predict boredom in the workplace. Workers who aren’t engaged in meaningful or fulfilling activities might also get uninterested in their jobs (Andel et al., 2022). The absence of stimulation from the outside world, the lack of learning opportunities, person–job misfit, the lack of job variety, and the lack of work overload have all contributed to job boredom.

Person–job fit theory states that when employees experience person–job mismatch, they experience job boredom. Those employees with surplus qualifications then their job requirements experience an unpleasant situation in the workplace and become dissatisfied with such an environment. Overqualified employees think their knowledge, skills, and abilities are underused and not fully utilized by the organization. In line with these arguments, there is a positive link between POQ and job boredom. Job boredom is also positively linked with work and skill underutilization (Ye et al., 2017). According to person–job fit theory, they are not making full use of their abilities, or their work environment hinders their abilities to do so. Previous studies show a link between job boredom and person–job fit incongruity. Still, research in this area is limited in the case of job boredom due to external stimulation (e.g., task variety, complexity). Research continually shows that workers’ opinions and attitudes at work are highly linked to perceived overqualification. Boredom may be caused by settings that do not fit an employee’s interests or job requirements (Fisher, 1993). Employees’ perceived overqualification or mismatch between their experience, education, competencies, and the demands of the work may be a contributing factor (e.g., individuals may believe that their previous work experience, expertise, and talents transcend the requirements of their current position) may have a role in determining the likelihood of experiencing job boredom (Kim et al., 2021).

*Hypothesis 1*: There is a positive relation between POQ and job boredom.

### Job Boredom and Counterproductive Work Behaviors

According to our findings, job boredom is an emotional pathway that leads to CWBs, which adds to the emotion-centered model of voluntary work behaviors (Bruursema et al., 2011). The workplace environment induces emotions, and such emotions cause actions that lead to behaviors. When an employee faces such unpleasant behaviors, it causes boredom. Boredom activates impulses to avoid such situations and search for coping behaviors that might reduce job boredom. As a result, to manage boredom, employees engage in counterproductive work behavior (He et al., 2020).

Voluntary actions that harm an organization or other persons are classified as “counterproductive” work behavior (Bennett and Robinson, 2000). The emotion-centered model of voluntary work behaviors states that stress-induced unpleasant emotions lead to CWBs (Spector and Fox, 2002). Fox et al. (2012) showed that boredom at work was linked to various CWBs, including mistreatment, undermining, and withdrawal behavior.

Limited study suggests that repetitive and monotonous activity may adversely affect employees and cause job boredom (Bruursema et al., 2011). Person–job fit theory states that people who complain about their jobs being boring are more likely to be absent from work, more likely to withdraw, less likely to be happy at work, and less likely to be productive (Spector and Fox, 2010). This research offers early verification for an association between job boredom and counterproductive work behavior. Although prior studies hypothesized links between job boredom and CWB, the mechanisms behind this association have not been rigorously studied. Workers could participate in counterproductive practices to “reduce boredom by producing a change of activity, reasserting personal freedom of choice, and giving the thrill of risking damage or discovery” (Fisher, 1993). Alternately, one may view the characteristics of professions that lead to boredom as a sort of occupational stressor that would be predicted to contribute to CWB (Cohen and Diamant, 2019). According to the job stressor model, employees typically respond to stressors by engaging in CWB (Spector and Fox, 2002). We argue that CWB is a technique for dealing with difficult or uninteresting circumstances.

*Hypothesis 2*: There is a positive relation between job boredom and CWB.

### Perceived Overqualification and Counterproductive Work Behaviors

We argue that unpleasant emotions due to POQ, is a kind of person–job misfit that impends employees comfort and encourage negative behavior, called CWB. Examining POQ and CWB link, we integrated the person–job fit theory. We argue that overqualified employees are more inclined to figure out what this mismatch implies to them cognitively (Biçkes et al., 2020). Employee’s cognitive reactions include lower self-esteem, anxiety, and frustration. When employee’s qualification and interest for a specific role does not match or underutilized creates work frustration and unpleasant situation. When employee’s needs are not fulfilled, they exhibit negative emotional reaction (Andel et al., 2022). Intention to avoid such unpleasant situation, overqualified employee’s response in the shape of anger, frustration toward unpleasant situation. Such emotional reaction may account for the potential association between perceived overqualification and CWB (Bruursema et al., 2011). Such negative emotional reactions to job misfit, overqualified employees adopt the “GO SLOW” approach—a protest by workers who deliberately work slowly to cause problems for their employers. Such employee’s speaks poorly about employees and organization to harm their reputation. Such employees express their negative feelings by not cooperating with their coworkers (Chambel et al., 2021a). Further, grounding on person–job fit theory, we link poor job fit with negative work emotions and support the argument that stress or frustration leads to CWB. We argue that performing such tasks and responsibilities from whom the employee is overqualified face frustration, leading to voluntary behaviors that harm organizations (CWB-O) or individuals working in the organizations (CWB-I) (Bennett and Robinson, 2000).

*Hypothesis 3*: Perceived overqualification is positively related to CWB.

### The Role of Job Boredom as a Mediator

In the case of overqualified individuals, negative emotions play an important part in determining their CWB (Liu and Wang, 2012). Khan et al. (2022a) used time-lag data on Pakistani workers to find that overqualified employees felt angry about their working conditions. When they were angry, they were more likely to affect themselves and their coworkers negatively. According to person–job fit theory, that overqualified persons participate in CWB because of negative emotional response to their profession (Luksyte et al., 2011). It was shown that only cynicism could properly mediate the relationship between POQ and CWB, highlighting the relevance of negative feelings in comprehending this focused relationship (Luksyte et al., 2011). Specifically (Deng et al., 2018) performed two multisource data studies. They found that when overqualified individuals misfit with the job requirements, they less socially participated, and this unpleasant sensation increased boredom. Boredom has gained recognition as a separate emotional state that impacts job behaviors and performance (van Hooff and van Hooft, 2014). Job boredom is a typical emotional response to POQ and may lead to CWBs (Liu and Wang, 2012). A similar finding has been made by Spector and Fox (2010), who claims that feeling bored at work due to POQ leads to CWB. CWB is more prevalent among overqualified workers (Erdogan et al., 2011). Overqualified individuals who experience a lack of enthusiasm at their place of employment may experience boredom due to the mismatch between their KSAs and their occupations which leads to higher involvement in CWB activities. The POQ study has shown that the CWB of overqualified persons may result job boredom (Spector and Fox, 2010; Liu and Wang, 2012). We hypothesized that the relationships between POQ and CWB would be mediated by job boredom.

*Hypothesis 4*: Job boredom mediates the relationship between POQ and CWB.

### Job Crafting as a Moderator

An overqualified employee can become easily bored and, eventually, an emotional drain due to the underutilization of their qualification. Person–job mismatch is the main and significant reason behind job boredom (Edwards, 1991). Previous research stated that POQ is significantly related to job boredom. According to Fisher (2018), a situation that does not fit employees needs and interest produce boredom. We argue that mismatch between knowledge, skill, and abilities or person–job incongruity and job requirements may induce frustration and leads to job boredom (Watt and Hargis, 2010). When employees perceive a disproportion between their abilities and the demands of their jobs, they may take proactive steps to improve the workplace’s pleasure and motivation by changing the work environment (Hu et al., 2022). Employees will be driven to adjust components of their work if the job does not fit their abilities or requirements (Wrzesniewski and Dutton, 2001). Job crafting is a job design strategy to divide job or work roles into different tasks to make them exciting and meaningful to reduce the positive effect between POQ and job boredom (Berg et al., 2013). Further, this approach is defined as “the self-initiated changes that employee’s make in their job demands and job resources to attain and optimize their personal (work) goals” (Tims et al., 2016). According to the theory of person–job fit, employees modify their jobs to better balance their needs and resources (Tims et al., 2012). In light of this process, proactive activities such as resource-seeking (structural and social), challenge-seeking, and demand reduction are part of job crafting (Wrzesniewski and Dutton, 2001). Feedback or information seeking, asking for guidance from peers, and maximizing professional autonomy are all resource-seeking activities. These behaviors may assist in making overqualified individual work more motivating and mobilizing additional resources to meet the demands of one’s employment to reduce job boredom (Yu and Jyawali, 2021). Performing new activities or accepting more duties are examples of actions associated with seeking difficulties. Overqualified individuals may participate in these practices to keep their motivation high and avoid becoming bored. By dividing one’s work into fascinating roles, overqualified individuals reduce the stress associated with one’s job by minimizing emotional, mental, and physical stresses (Petrou et al., 2012). As a result, when their work is customized for them *via* “job crafting,” employees have a more favorable perception of the relevance of their employment (Wrzesniewski et al., 2013).

Persons who make their employment enjoyable reported more excellent person–job fit. Higher levels of demands–abilities fit were associated with better levels of work satisfaction and low level of job boredom. However, this early evidence suggests that job crafting may be a useful strategy to code job boredom. In order to acquire meaningfulness in contemporary work environments, it is necessary for overqualified individuals to have the ability to craft the job (Wrzesniewski et al., 2013). It is possible to improve the quality of one’s work-life by modifying one’s work environment to better suit one’s personality and work goals (Tims et al., 2016). Workplace boredom may be reduced or eliminated by giving employees the freedom to modify their professions, allowing them to create meaningful and engaging experiences for themselves *via* job crafting (Berg et al., 2013).

*Hypothesis 5*: Job crafting moderates the positive association between perceived overqualification and job boredom.

**Figure 1:**
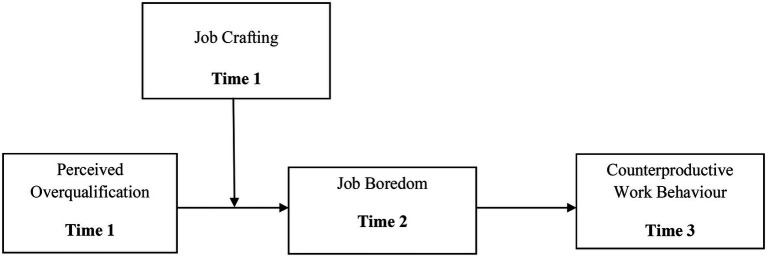
Hypothesized moderated mediation model.

We examine job crafting as a preemptive individual approach to enhance person–job congruity, which may reduce the interaction effect of POQ and job boredom on CWB. Examining job crafting as a proactive employee action that aid in detecting work design or self-initiated strategies through which boredom may be reduced, and ultimately CWB can be reduced. Earlier, we argued that POQ is positively linked with the CWB *via* job boredom. Consequently, perceived overqualification leads to job boredom, which was shown to be the major driver of counterproductive behavior in our study. Furthermore, we stated that employees who engage in job crafting will acquire more fabulous sentiments of motivation to make their jobs more fascinating, will face less job boredom in reaction to perceived overqualification, and will be less likely to engage in counterproductive work behavior. Thus, we hypothesized that the positive link between perceived overqualification and job boredom would be lower for those who are more involved in job crafting. Therefore, we hypothesize that (Figure 1):

*Hypothesis 6*: The indirect link between perceived overqualification and counterproductive work behavior *via* job boredom is stronger at higher than at lower levels of job crafting.

## Methodology

### Sample and Procedure

Data were collected from five textile companies located in Khyber Pakhtunkhwa Pakistan. This industry is selected on the basis of its significance and contribution to the economy. Pakistan is the eighth biggest exporter of textile items in Asia, according to the World Trade Organization. It is the fourth biggest producer and third largest consumer of cotton in the world. It accounts for 46 percent of the overall manufacturing sector’s output and employs 40 percent of the nation’s work force. Only 5% of the total number of textile firms are publicly traded on the stock market. In the long term, any inconsistency or carelessness in catering criteria would have severe negative consequences for the whole population. The inability to provide a suitable working environment for all workers, as well as the failure to address individual employee needs, will eventually result in a reduction in the overall quality of the job, resulting in a loss to the whole organization. The authors contacted the participants through professional and personal sources. The HR department of the concerned organization were contacted for help to take approval from the top management. The study’s purpose was communicated with the organization’s members. After obtaining official approval, we distributed questionnaires among employees, assured that the data collection was voluntary, and guaranteed the respondents’ privacy. The questionnaires were coded adequately due to the time-lag study (to match T1, T2, and T3). The respondents were asked to place their questionnaires in sealed envelopes. The envelopes were gathered by the authors. To avoid common method biases (Podsakoff et al., 2012), the survey was conducted in three phases with a gap of 5 weeks. Podsakoff et al. (2012) suggest that the gap should be neither too short nor long. If the gap between data collection is too long, participant attrition happen. Similarly, if the length is too short of the data collection, memory effects may magnify the relationship between variables in an unnecessarily inflated manner (Dormann and Griffin, 2015). Thus, it was decided to maintain a gap of 5 weeks is the best choice (Dormann and Griffin, 2015). In phase 1, we collected data from 412 respondents out of 463 respondents regarding POQ, job crafting, and demographics and we received responses (88.98%). After 5 weeks gap, in phase 2, we contacted the same respondents to complete the second phase questionnaire regarding job boredom and received 364 responses. In phase 2, we received the response rate was (78.61%). After 5 weeks, we collected data for CWB for those who participated in the second phase. Three hundred and twenty-six (326) responses were received in phase 3, the participation rate was (70.41%). After proper scrutiny we removed nine questionnaires due to missing values. Finally, we have sample of 317 employee’s data for analysis. The following are their demographic characteristics: The average age of the workers was 32.0 years, and 68.0% of the workforce were male. The respondent’s education indicate that 87.2% of workers held a master’s degree or more.

## Measures

### Perceived Overqualification

The 9-item scale was used to assess overqualification (Maynard et al., 2006). Example of items are (e.g., “I have more abilities than I need to do my job;” *α* = 0.75).

### Job Boredom

To measure job boredom we used 6-item Dutch boredom scale, developed by Reijseger et al. (2013). A sample item is “I have felt bored at my job.” The reliability of this study was *α* = 0.85.

### Job Crafting

The job crafting scale has 15 items used to measure employee job crafting (Tims et al., 2012). “Increasing structural job resources (*α* = 0.76), increasing social resources (*α* = 0.77), and increasing challenge job demands (*α* = 0.75)” were each assessed by five items.

### Counterproductive Work Behavior

CWB was measured by using a (Bennett and Robinson, 2000) 19-item scale. The scale contains two dimensions: CWB against the coworkers/individuals (CWB-I; 7 items) and CWB against the organization (CWB-O; 12 items). A sample item of CWB-I is “Said something hurtful to someone at work,” and a sample item of CWB-O is “Taken an additional or longer break than is acceptable at your workplace.” Higher scores represent a higher level of counterproductive behavior. The Cronbach’s α of CWB-I and CWB-O for the current data set was 0.92 and 0.94, respectively.

#### Control Variables

In the present research, the relationship between age and job boredom was negative and statistically significant (*r* = −0.032, p. 01). Younger employees reported greater job boredom than their older counterparts, as shown by earlier research (Harju et al., 2014). As a result, we chose to include age as a controlling factor in our study.

## Results

### Descriptive Statistics

Table 1 displays the means, standard deviations, correlations, and scale reliability. As demonstrated in Table 1, all study variables exhibited a satisfactory level of internal consistency, which was considered acceptable. The correlations between study variables were in the predicted direction, and all study variables had an acceptable degree of internal consistency (*α* > 0.70). The POQ was positively related to job boredom (*r* = 0.24, *p* < 0.01) and CWB (*r* = 0.31, *p* < 0.05). In addition, employees’ job boredom was positively related to CWB (*r* = 0.21, *p* < 0.01).

**Table 1:** Correlations, mean, standard deviation, and reliability.

Variables	Mean	SD	1	2	3	4	5	6	7	8
1. Gender	1.39	0.48								
2. Age	2.83	0.95	0.247							
3. Service	1.72	0.90	0.219	0.700						
4. Education	2.89	0.51	0.042	0.048	−0.063					
5. POQ	4.02	0.34	0.022	0.002	0.017	0.024	**(0.82)**			
6. CWB	3.58	0.54	−0.047	−0.078	−0.067	0.045	0.314^**^	**(0.79)**		
7. Job boredom	3.56	0.80	0.027	−0.032^*^	0.044	0.061	0.244^**^	0.216^**^	(0.84)	
8. Job crafting	1.73	0.36	0.024	0.040	0.039	0.020	0.033	−0.062	−0.072	**(0.87)**

* Correlation is significant at the 0.05 level (2-tailed).

** Correlation is significant at the 0.01 level (2-tailed). Bold values are Cronbach’s alpha values.

### Model Measurement

To determine the questionnaire’s validity and reliability, we used CFA. The validation test shows that all indicators had a loading factor value > 0.5, indicating that they were acceptable (see Table 2). Prior to evaluating the hypotheses, Anderson and Gerbing (1988) recommended assessing the construct validity of variables. Confirmatory factor analysis (CFAs) using AMOS 18.0 confirmed the distinctness of our model’s POQ, job boredom, job crafting, and CWB constructs. The results showed in Table 3, the four-factor model provided a good fit with fit indices in acceptable range (*χ*2 = 313.24, *df* = 69, GFI = 0.92, CFI = 0.91, TLI = 0.90, RMSEA = 0.05). For each construct, the correlation between the constructs is shown in Table 2, which is smaller than the square root of the AVE in each of the dependent and independent constructs. Thus, the discriminant validity of the model was effectively supported by this research.

**Table 2:** Factor loading’s.

Items	CR	AVE	Loadings
POQ	0.93	0.63	
Item 1			0.80
Item 2			0.73
Item 3			0.78
Item 4			0.87
Item 5			0.83
Item 6			0.79
Item 7			0.82
Item 8			0.79
Item 9			0.75
Job crafting	0.96	0.62	
Item 1			0.76
Item 2			0.78
Item 3			0.82
Item 4			0.78
Item 5			0.69
Item 6			0.81
Item 7			0.77
Item 8			0.76
Item 9			0.83
Item 10			0.78
Item 11			0.82
Item 12			0.83
Item 13			0.77
Item 14			0.76
Item 15			0.89
Job boredom	0.92	0.68	
Item 1			0.81
Item 2			0.76
Item 3			0.83
Item 4			0.84
Item 5			0.88
Item 6			0.84
CWB	0.95	0.60	
Item 1			0.79
Item 2			0.77
Item 3			0.84
Item 4			0.77
Item 5			0.82
Item 6			0.73
Item 7			0.72
Item 8			0.78
Item 9			0.77
Item 10			0.74
Item 11			0.81
Item 12			0.87
Item 13			0.71

**Table 3:** Measurement model comparison (*N* = 317).

Model’s	RMSEA	GFI	CFI	TLI	*X*^2^(*df*)	Δ*X*^2^ (*df*)
Model-4 (POQ, CWB, JB, and JC)	0.05	0.92	0.91	0.90	313.24^***^(69)	–
Model-3 (POQ, CWB, JB, and JC)	0.11	0.71	0.87	0.81	421.12^***^(73)	289.23^***^(3)
Model-3 (POQ, JB, JC, and CWB)	0.18	0.56	0.55	0.77	524.41^***^(85)	346.11^***^(4)
Model-2 (POQ, JB, CWB, and JC)	0.21	0.47	0.47	0.57	813.45^***^(86)	422.14^***^(4)
Model-1 (POQ, JB, CWB, andJC)	0.26	0.31	0.33	0.41	927.32^***^(88)	653.21^***^(7)

*** Correlation is significant at the 0.001 level (2-tailed).

### Hypotheses Testing

#### Direct and Mediation Effects

As shown in Table 2, hierarchical linear regression was applied to analyze the main effects of the study, and (Hayes, 2017) PROCESS macro was used for mediation, moderation, and moderation mediation. First, we examined the association between POQ and job boredom, and the results indicate that there is a positive relationship exists between these two variables (Model 1: *B* = 0.571, *p* < 0.000), thus supporting hypothesis 1. Second, we examined the direct link between job boredom and CWB, and the results indicates a positive association between job boredom and CWB (Model: 4 *B* = 0.293, *p* < 0.000), supporting hypothesis 2. Third, the results show that POQ positively affects CWB, supporting our hypothesis 3 (Model 4: *B* = 0.653, *p* < 0.000). Mediation analyses were performed using Hayes (2017) PROCESS macro model 4 (see Table 2). Job boredom partially mediated the link between POQ and CWB (indirect effect = 0.293, SE = 0.075, 95% CI = 0.1437, 0.4426; direct effect = 0.36, SE = 0.049, 95% CI = 0.2634, 0.4567), thus supporting hypothesis 4.

#### Moderation Effects

**Figure 2:**
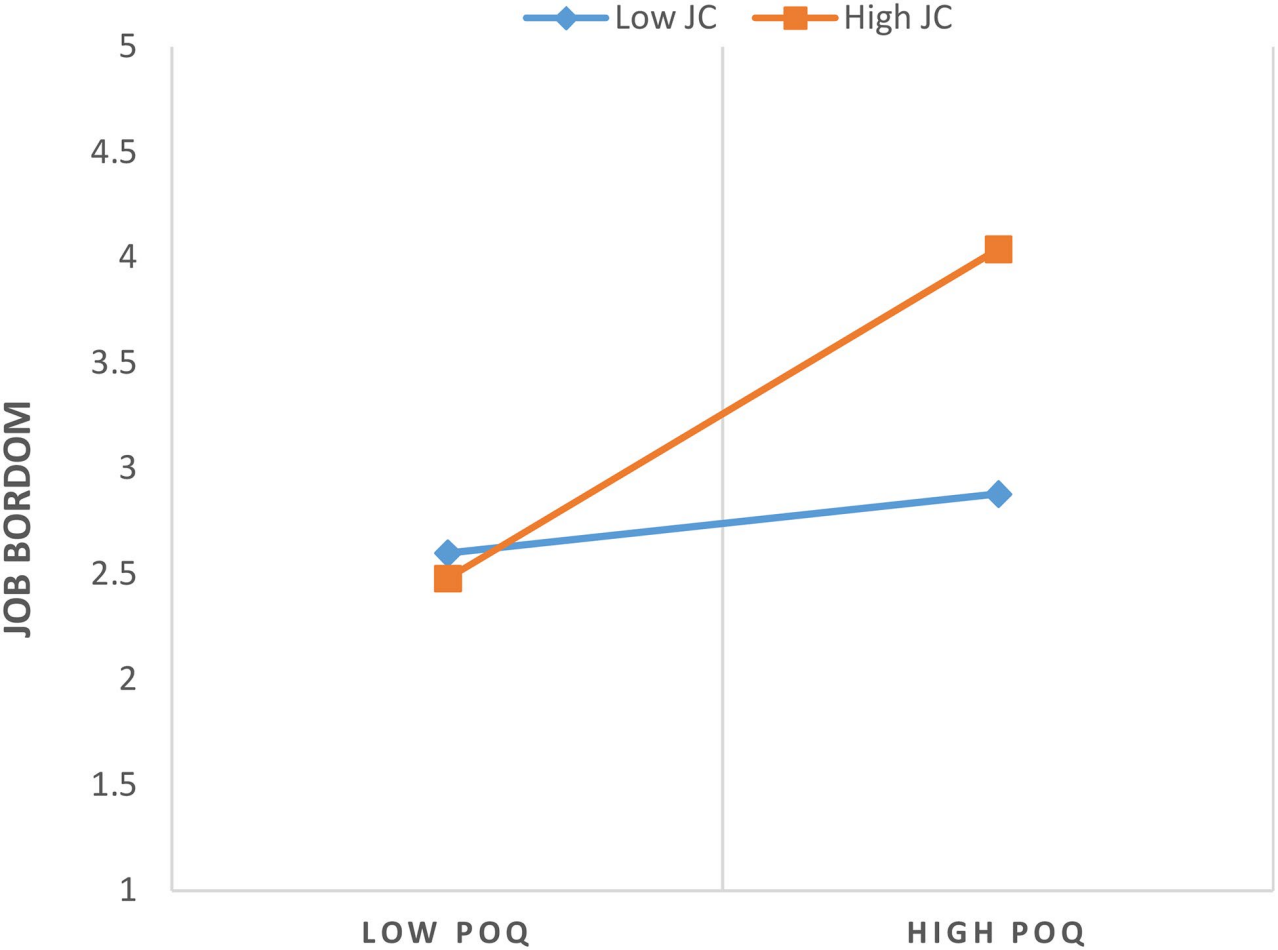
Interactive effect of POQ and job crafting on CWB.

For moderation analyses, we used Hayes (2017) PROCESS macro model 1. Hypothesis 5 stated that job crafting significantly moderates the positive relationship between POQ and job boredom as shown in Table 4 (Model 2: *B* = −0.093^*^, SE = 0.4288, *p* < 0.05, 95% CI = −1.9575, − 0.2701). Figure 2 shows the interaction effect, which we displayed to make it easier to understand. Job crafting was measured using basic slopes tests (i.e., + 1 and − 1 SD from the mean; Aiken et al., 1991). Our findings demonstrated a significant link between POQ and job boredom when an employee’s job crafting was high (simple slope = 0.94, SE = 0.1995, *p* < 0.000); as compare to when it was low (simple slope = 0.16, SE = 0.2039, *p* > 0.005). These findings support the pattern stated in hypothesis 5 and illustrate that employees’ perceived overqualification and employees’ job boredom relation will be weak by practicing job crafting.

**Table 4:** Hierarchical linear regression results.

Variables	Job boredom			Counterproductive work behavior		
	Model 1	Model 2	Model 3	Model 4	Model 5	Model 6
Age	0.03	0.05	0.04	0.03	0.04	0.03
Gender	−0.07	0.04	0.03	0.04	−0.11	−0.14
Education	−0.03	0.02	−0.11	0.03	−0.06	−0.03
Service	0.09	0.06	−0.01	0.02	0.03	0.01
POQ	0.571^***^			0.653^***^		
Mediator				0.551^***^		
Job boredom					0.293^***^	
Moderator						
Job crafting		−0.093^*^				
Interaction effect						
POQ × job crafting						−1.1138^*^
*R* ^2^	0.15	0.17	0.14	0.18	0.15	0.17
*F*	21.13^***^	24.01^***^	31.05^***^	27.08^***^	12.01^***^	31.07^***^

* Correlation is significant at the 0.05 level (2-tailed).

*** Correlation is significant at the 0.001 level (2-tailed).

#### Moderation Mediation Examination

In an integrated manner, we investigated the moderation mediation analysis of hypothesis 6 (One SD above and below the mean of a moderator). It was shown that the moderated mediation model, which included the outcome variable of CWB, was significant when job crafting was high (conditional indirect effect = 0.50, SE = 0.12, 95% CI = 0.2700, 0.7519), as compare to when job crafting was low (conditional indirect effect = 0.08, SE = 0.11, 95% CI = −0.1283, 0.3053). Therefore, the index of moderated mediation was statistically significant (Index = −0.5716, SE = 0.24, 95% CI = −1.0512, − 0.0934), confirming hypothesis 6 (see Table 5).

**Table 5:** Moderated mediated results for POQ and CWB across levels of job crafting.

Mediator	Level	Conditional indirect effect	SE	LLCI	ULCI
Job boredom	Low(–1SD)	0.0830	0.1103	−0.1244	0.3036
	High(+1SD)	0.5018	0.1249	0.2700	0.7600
	Difference	0.4188	0.0146	0.3944	0.4564

Moderator values are the mean and ± 1 SD, LLCI lower limit 95% confidence interval, ULCI upper limit 95% confidence interval.

## Discussion

Based on person–job fit theory, we found that perceived overqualification positively affected job boredom and CWB. In addition, the link between overqualification and CWBs is mediated by boredom at work. Furthermore, job crafting moderates the positive association between overqualification and job boredom. Moreover, we found that job crafting interacted with perceived overqualification, affecting job boredom and counterproductive work behavior (Yu and Jyawali, 2021). The study results showed that job crafting weakens the effect of job boredom on counterproductive work behavior after job crafting (Schreurs et al., 2020). As a result of our research, substantial theoretical and practical implications been identified.

### Contributions to Theory and Research

First, this study examined job boredom as an outcome of perceived overqualification. In accordance with theoretical support job boredom acts as a mediating factor between perceived overqualification and CWBs. In other words, it adds to our understanding of how this connection is made and furthers prior studies that have identified factors such as exhaustion, anger, and breakdown in the psychological contract due to overqualification (Khan et al., 2022b). According to (Andel et al., 2022), boredom at work leads to resentment, which causes individuals to lash out in retaliation toward the thing or person they feel is the cause of their withdrawal behavior (Howard et al., 2022). Overqualification-induced job boredom has been linked to the CWBs in our research. This is an important finding, to now the association between job boredom and CWBs has never been systematically investigated in the context of perceived overqualification. Our study add to the findings of Erdogan and Bauer (2021), who found that job boredom is associated with subjective well-being and career satisfaction, which, in contrast to boredom-driven reactions, are positive and low-arousal experiences (Harju et al., 2016).

Second, according to our research, workers who think overqualified are more likely to believe they are entitled to greater pay (Erdogan et al., 2018). It has been reported that the perception of overqualification develops by comparing coworkers and those who are not overqualified (Hu et al., 2015; Khan et al., 2021). Such perceptions make overqualified employees bored at their jobs compared with their notable coworkers. Grounding on person–job fit theory (Edwards, 1991), when people start comparing their past or future career opportunities and comparing their KSA and needs, they start feeling bored. This study further explains the overqualification phenomenon and underpins person–job fit theory as a new addition. Working in a boring environment may be a multifaceted state of well-being with various antecedents. However, most research on the topic focuses on the nature of the job as a predictor (e.g., routine, repetitive duties, lack of variety) and the effects, both positive and negative (Harju et al., 2016; Khan et al., 2022b). The results of this study also corroborate earlier qualitative findings that support our novel study and insert to the body of knowledge in the area of POQ (Liu and Wang, 2012; Erdogan and Bauer, 2021).

Third, overqualified persons demand career planning, tasks, responsibilities, status, and promotions that are appropriate for their qualifications, and the opportunity to advance in their careers (Gong et al., 2021; Zada et al., 2022a). Therefore, when put in jobs for which they believe they are overqualified, they suffer more boredom. The perception of overqualification is more prevalent among people who do not engage in job crafting since there is a wider disparity between one’s existing position and one’s desired status. In accordance with this rationale, we found that job crafting motivates people to evaluate how they behave, engage, and feel regarding their work and to redesign and personalize parts of their job to increase engagement, job satisfaction, tolerance, and a desire to overcome boredom.

Fourth, by identification job crafting as a boundary condition, our research adds a significant amount of contribution to perceived overqualification and its consequences. There are various individual-level moderating variables that Liu and Wang (2012) identify in their analysis of research on perceived overqualification, including justice sensitivity and empowerment. Our findings validated their model because we found an individual-level moderator. We add to the literature by demonstrating that job crafting, as a self-driven personality attribute, is a moderator that overcomes boredom. In their model, this feature has not yet been discussed. It may be a useful inclusion as a boundary condition for the interaction between overqualification, job boredom, and CWBs. We interpret these findings as showing that job crafting enables people to fully apply their talent and abilities to their jobs. Consequently, they play an essential role in success business. The findings indicate that although job crafting is seen as a positive personal trait, it may also have the additional advantage of increasing people’s likelihood of experiencing emotions of pleasure in their work.

### Practical Implications

Our study’s findings supported HR professionals’ concerns that overqualification leads to negative attitudes and behaviors (Khan et al., 2022a). However, this is not the case in rejecting an overqualified person. In return, organizations may expect higher expectations. The management of overqualified employees is important for organization. When appropriately managed, it becomes an organization’s asset (Erdogan et al., 2011). Based on this research, organizations with a good number of overqualified individuals may benefit from overcoming CWB.

First, the organization should work on employee’s self-esteem and develop strategies that may help employee’s self-esteem. When employees have healthy self-esteem, they feel good about themselves and organizations. Organizational self-esteem may be raised by changes in organizational structure (e.g., job design), better interpersonal connections, and creating work environments that encourage maximum output (Pierce and Gardner, 2004; Zada et al., 2022b). Therefore, we believe that giving overqualified individuals greater job autonomy will help them boost their organizational self-esteem since they will be able to adjust their employment to make them more productive proactively. Overqualified individuals may be involved in decision-making, which will positively impact their self-esteem as a result of their association with the organization (Pierce and Gardner, 2004; Khan et al., 2022a). This will help them generate suggestions on improving the current processes by using their underutilized qualifications, as well as management that places a high value on their credentials and that they care about them, which is critical. Additionally, the organization might invest more time and effort into socializing its overqualified personnel so that others are more familiar with these employees and are more likely to contact them for professional guidance or mentorship. Overqualified employees will be able to put their excess talents and abilities to use for organization and personal developments. These measures are likely to boost overqualified employees’ sense of self-worth while decreasing their proclivity to participate in CWB.

Second, regarding the mediating function of job boredom toward employment circumstances in the overqualification–CWB relationship, we propose that organizations attempting to reduce the CWB of their overqualified personnel should use supporting techniques that effectively reduce boredom (Gibson and Callister, 2010). When it comes to person–job mismatch concerns, overqualified workers might be encouraged to take the initiative and speak out with their supervisors. Overqualified people are likely to redirect their boredom in such a circumstances, notably by identifying ways to put their underutilized qualifications to use in the workplace. Additionally, by offering a realistic work preview, organizations may prevent their overqualified employees from becoming bored with their job. When overqualified individuals get an impartial evaluation of how well their credentials fit job criteria, they may come to trust their employers and their hiring organizations.

Third, considering that this honesty helps fulfil the psychological contract, we believe that it will likely reduce work boredom when the employee has a high degree of prior information about the task at hand. To assist overqualified employees in managing their anger, employee support programmes should be created, including the promotion of forgiveness and encouragement of a problem-focused coping style. They can overcome the negative repercussions of being angry at a job (e.g., CWB) and instead concentrate on strategies to improve their poor person–work fit. Although these tactics are certainly successful for all overqualified individuals, they may be especially helpful in reducing negative emotions regarding overqualification among those who do well in job crafting. Organizations may identify overqualified individuals who may be too sensitive to person–job mismatches by enhancing selection techniques with personality evaluation. To minimize negative emotional and cognitive responses to overqualification, they should be prioritized in receiving techniques. Hiring employees who are overqualified for their position is often presented negatively, with the general belief being that they will rapidly become bored in their position and leave the organization. This is not always the case, but it is advised that more studies be conducted on workers who other overqualified persons surround since they are more likely to succeed. According to our findings, the most effective way to assess employee attitudes is to surround them with peers of comparable qualities.

### Strengths, Limitations, and Future Directions

Our study had several positive aspects. We used a time-lagged research design, that allowed us to reduce several prevalent risks in our study. Our study, conducted in a single organization that operated in various sectors, enabled us to guarantee that human resource policy and organizational culture remained consistent while ensuring that perceptions of overqualification and job insecurity varied from one industry to the next. When interpreting the results, it is essential to keep in mind that several challenges, such as self-report surveys, may lead to common technique bias. Due to its widespread usage in academic literature and pattern matching as a survey methodology, self-reporting may be appropriate for certain studies (Spector, 1994). First, the small sample size of our study is a significant drawback of our research. Because of the time-lag design, there was significant subject attrition, resulting in a small sample size. While the findings supported certain assumptions, their generalizability of the findings might be improved by utilizing a larger sample size.

The second limitation is that this research was conducted individually; nevertheless, examining how jobs are created at the group, department, and organization levels is important in future. Third, in certain cases, new employees may be regarded as overqualified, but long-term staff may not be. Once hired, employees may feel they are overqualified for their work, or they may start to realize this over a period of time in the position after starting. A person who feels overqualified in a shorter amount of time may find it challenging to succeed in job crafting “(i.e., I see that I am overqualified, but it’s not going to take long, so I’ll just wait it out).” Job crafting, on the other hand, maybe more likely be used by someone in a long-term overqualification scenario because they will have less opportunity to locate a better fit “(i.e., I see that I am overqualified and I’m going to be stuck in this job, so I’d better find ways to make it more palatable).” In this study, the effects of situational factors on workers’ reactions to emotions of overqualification were not examined. Further studies might benefit from an examination of new workers as well as the molding and development of cooperative tendencies.

## Conclusion

The current research based on person–job fit theory examined a moderation mediation model describing how POQ effects knowledge hiding. Furthermore, we examined the mediating role of job boredom between POQ and knowledge hiding. Moreover, we used job crafting as a boundary condition that effects the strengths of the indirect effect of POQ on knowledge hiding. Our results showed that the POQ indirectly impact knowledge hiding through job boredom. In addition, job crafting buffered the relation between POQ and the mediator (i.e., job boredom) and the indirect relationship between perceived overqualification and knowledge hiding.

## Data Availability Statement

The raw data supporting the conclusions of this article will be made available by the authors, without undue reservation.

## Author Contributions

JK, AA, and IS contributed to the conception and design of the study and wrote the first draft of the manuscript. JK organized the database and performed the statistical analysis. JK, AV-M, and NC-B wrote sections of the manuscript. All authors contributed to the article and approved the submitted version.

## Conflict of Interest

The authors declare that the research was conducted in the absence of any commercial or financial relationships that could be construed as a potential conflict of interest.

## Publisher’s Note

All claims expressed in this article are solely those of the authors and do not necessarily represent those of their affiliated organizations, or those of the publisher, the editors and the reviewers. Any product that may be evaluated in this article, or claim that may be made by its manufacturer, is not guaranteed or endorsed by the publisher.
